# Supplementary material for: Basal ganglia output dynamically controls skilled forelimb kinematics in real time
Source: bioRxiv. 2026 Mar 11:2026.03.09.710687. Preprint. [Version 1] doi: 10.64898/2026.03.09.710687 (PMC13060906; doi:10.64898/2026.03.09.710687)
Supplement: Supplement 1 [file media-1.pdf]

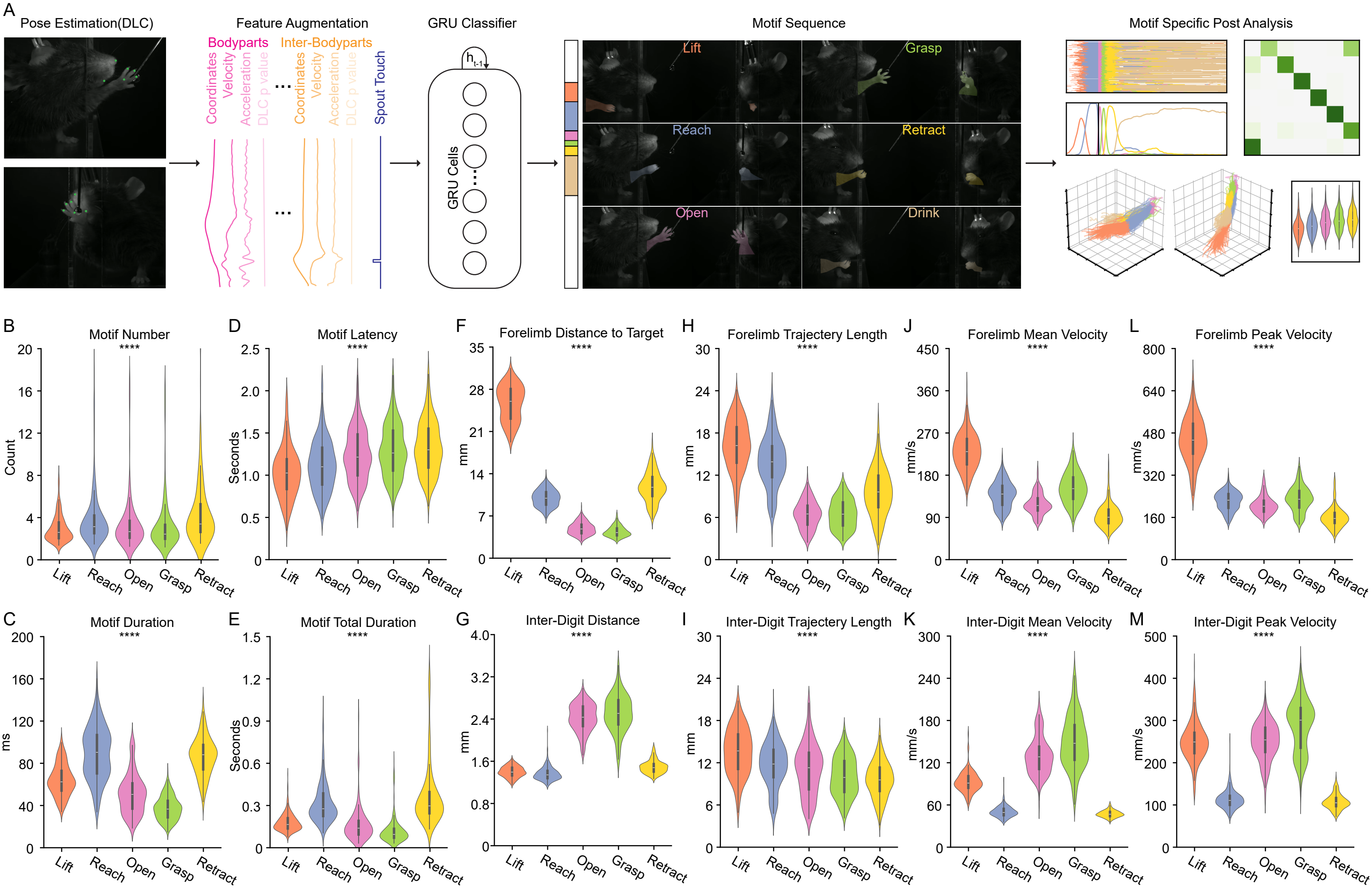
A
Pose Estimation(DLC)
Feature Augmentation
Bodyparts
Inter-Bodyparts
Coordinates
Velocity
Acceleration
DLC p value
Spout Touch
GRU Classifier
GRU Cells
Motif Sequence
Lift
Grasp
Reach
Retract
Open
Drink
Motif Specific Post Analysis
B
Motif Number
Count
C
Motif Duration
ms
D
Motif Latency
Seconds
E
Motif Total Duration
Seconds
F
Forelimb Distance to Target
mm
G
Inter-Digit Distance
mm
H
Forelimb Trajectery Length
mm
I
Inter-Digit Trajectery Length
mm
J
Forelimb Mean Velocity
mm/s
K
Inter-Digit Mean Velocity
mm/s
L
Forelimb Peak Velocity
mm/s
M
Inter-Digit Peak Velocity
mm/s
****
Lift Reach Open Grasp Retract

**Figure S1. Establishment of a GRU (Gated recurrent unit)-based classifier for automated reaching sequence segmentation and motif-specific analysis, related to Figure 1.**

**(A)** Schematic of the behavioral analysis pipeline. DeepLabCut (DLC) was used to track six key body parts (digits 2–4, paw, mouth, and nose). Extracted coordinates were used to calculate pairwise inter-bodyparts coordinate and then were augmented with derived kinematic features, including velocity, acceleration. The augmented features were used as input to a GRU classifier for automated motif segmentation and subsequent analysis.

**(B–M)** Quantification of temporal and kinematic parameters across action motifs from a large dataset (n=24498 control trials, 251 sessions from 28 mice). Violin plots display the full data distribution density; internal box plots represent the median (center line) and the interquartile range. Parameters are grouped into: **(B–E)** temporal statistics (motif count, duration, latency, and total duration); **(F–I)** coordinate related metrics (distance to target, inter-digit distance, and trajectory travelled length); and **(J–M)** velocity related metrics (mean and peak velocities for the forelimb and inter-digit spread). All quantified parameters exhibited significant variation across motifs (Friedman test, ****$p < 0.0001$)

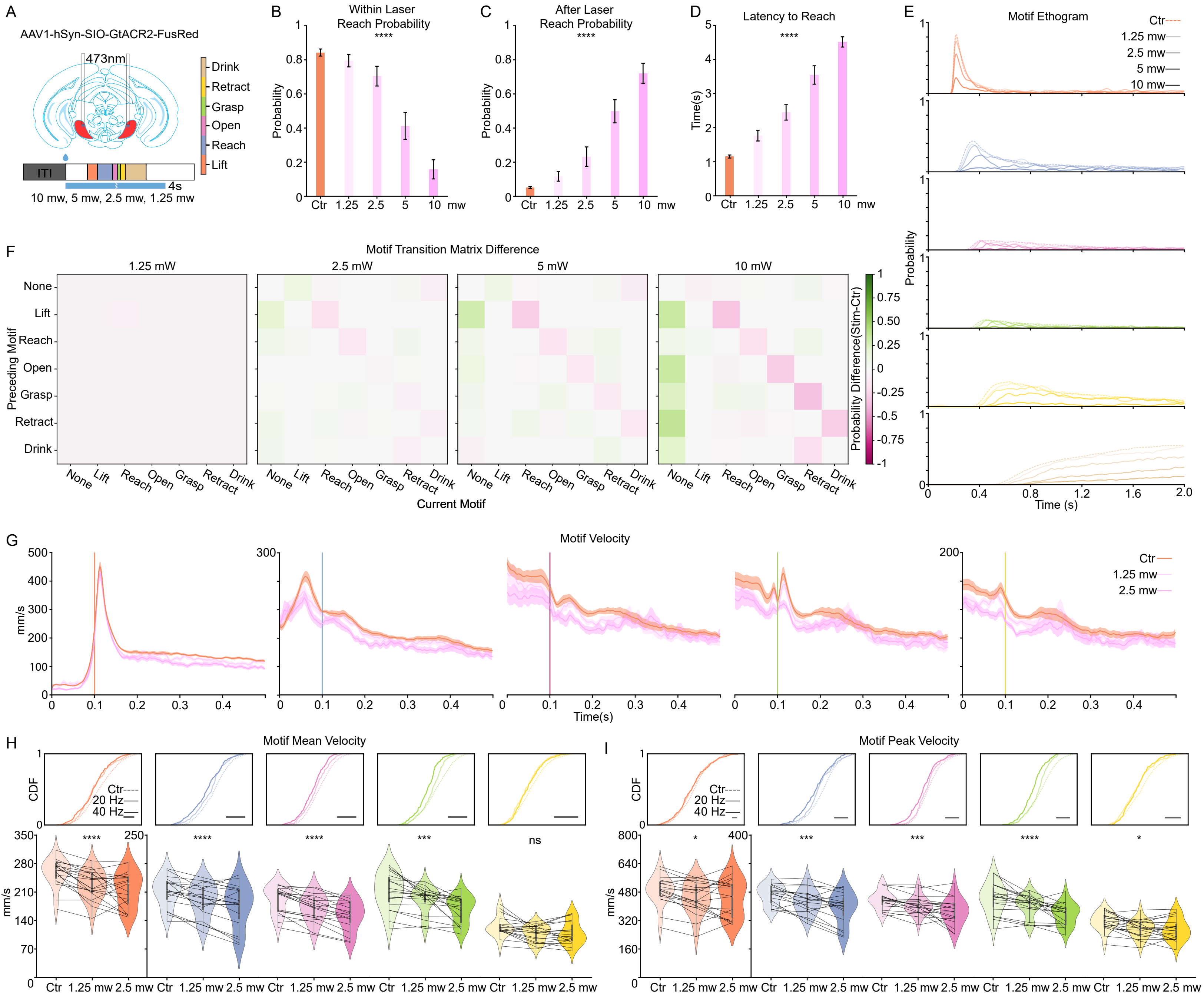

A
AAV1-hSyn-SIO-GtACR2-FusRed
473nm
Drink
Retract
Grasp
Open
Reach
Lift
ITI
4s
10 mw, 5 mw, 2.5 mw, 1.25 mw
B
Within Laser Reach Probability
****
Probability
Ctr 1.25 2.5 5 10 mw
C
After Laser Reach Probability
****
Probability
Ctr 1.25 2.5 5 10 mw
D
Latency to Reach
****
Time(s)
Ctr 1.25 2.5 5 10 mw
E
Motif Ethogram
Ctr
1.25 mw
2.5 mw
5 mw
10 mw
Probability
Time (s)
F
Motif Transition Matrix Difference
1.25 mW
2.5 mW
5 mW
10 mW
None
Lift
Reach
Open
Grasp
Retract
Drink
Preceding Motif
Current Motif
Probability Difference(Stim-Ctr)
G
Motif Velocity
mm/s
Time(s)
Ctr
1.25 mw
2.5 mw
H
Motif Mean Velocity
CDF
Ctr
20 Hz
40 Hz
mm/s
****
****
****
***
ns
Ctr 1.25 mw 2.5 mw
I
Motif Peak Velocity
CDF
Ctr
20 Hz
40 Hz
mm/s
*
***
***
****
*
Ctr 1.25 mw 2.5 mw

**Figure S2. Intensity-dependent modulation of forelimb reaching behavior by SNr photoinhibition, related to Figure 2.**

**(A)** Schematic of the experimental strategy. An inhibitory opsin (AAV1-hSyn-DIO-GtACR2-FusRed) was injected into the SNr and an optical fiber was implanted above the region. Photoinhibition was delivered using a 473 nm blue laser at four different intensities (1.25 mW, 2.5 mW, 5 mW, 10 mW), applied randomly across trials starting at water delivery and lasting for 4 seconds. The motif color map is provided (inset).

**(B–D)** Quantification of reach initiation dynamics: **(B)** Probability of reaching during laser delivery (Within Laser Reach Probability); **(C)** Probability of reaching following laser cessation (After Laser Reach Probability); and **(D)** Latency to Reach. (n = 21 sessions, 7 mice; Friedman test).

**(E)** Motif ethogram aligned to the first Lift motif, stratified by inhibition intensity (n = 21 sessions, 7 mice).

**(F)** Motif transition matrix difference maps, stratified by laser intensity. Each matrix displays the difference in transition probability (Inhibition - Control). n = 21 sessions, 7 mice.

**(G)** Forelimb velocity profiles (mean ± SEM) aligned to the onset of Lift, Reach, Open, Grasp, and Retract motifs (left to right). Only low-intensity trials (1.25 mW and 2.5 mW) are shown, as higher intensities blocked the majority of reaching attempts (n = 19 sessions, 7 mice).

**(H and I)** Quantification of motif Mean Velocity and Peak Velocity across different laser intensities. Top panels show the Cumulative Distribution Function (CDF) plots; bottom panels show the violin plots. (n = 19 sessions, 7 mice; Friedman test).

Data in bar plot are shown as mean ± SEM. Dots in the bar and violin plots represent individual session means. Violin plots display the full data distribution density; internal box plots represent the median (center line) and the interquartile range. ns no significance, *$p < 0.05$, **$p < 0.01$, ***$p < 0.001$, ****$p < 0.0001$.

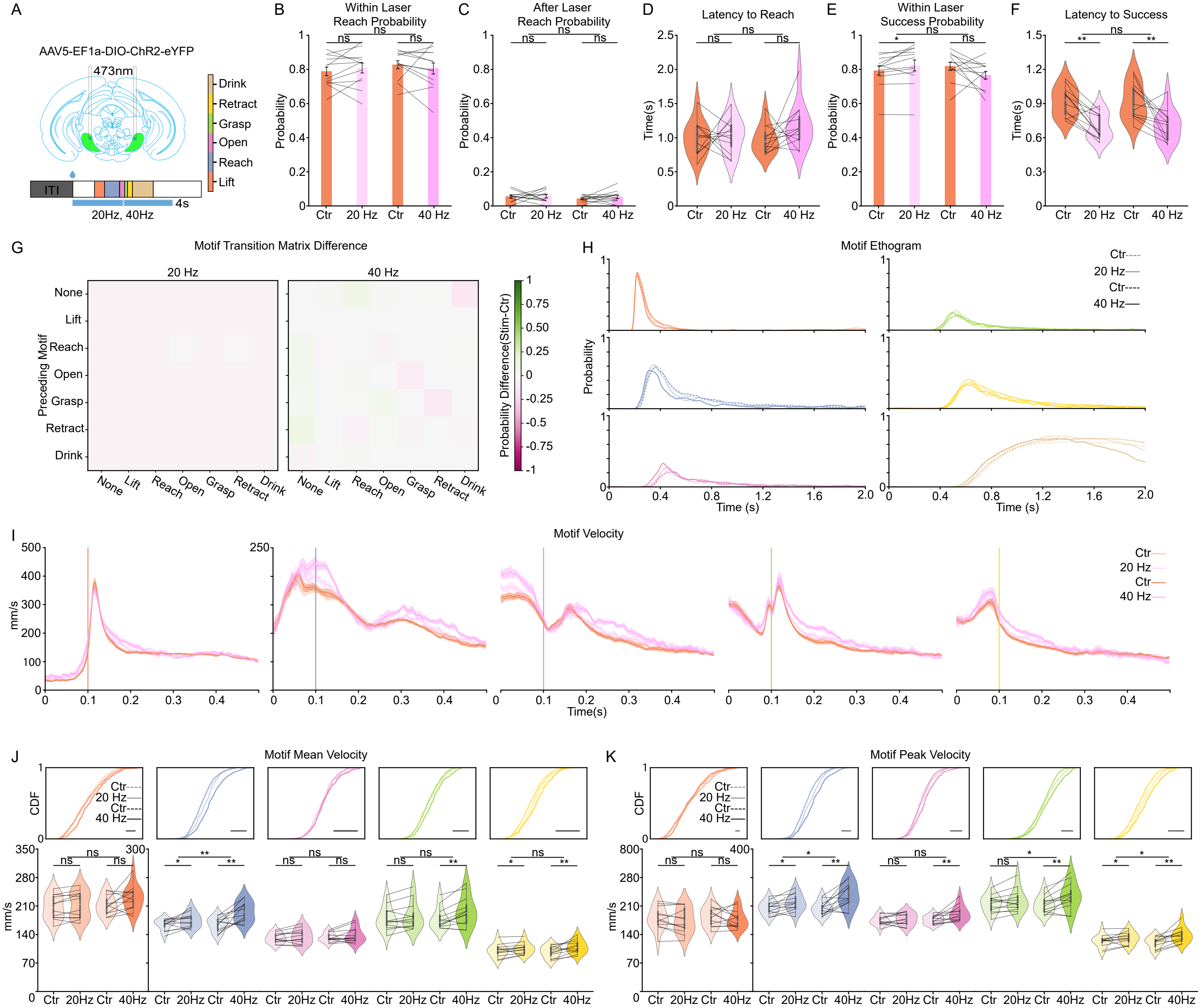
A
AAV5-EF1a-DIO-ChR2-eYFP
473nm
Drink
Retract
Grasp
Open
Reach
Lift
ITI
4s
20Hz, 40Hz
B
Within Laser
Reach Probability
C
After Laser
Reach Probability
D
Latency to Reach
E
Within Laser
Success Probability
F
Latency to Success
Probability
Time(s)
Ctr
20 Hz
40 Hz
ns
*
**
G
Motif Transition Matrix Difference
20 Hz
40 Hz
Preceding Motif
None
Lift
Reach
Open
Grasp
Retract
Drink
Probability Difference(Stim-Ctr)
H
Motif Ethogram
Time (s)
I
Motif Velocity
mm/s
Time(s)
J
Motif Mean Velocity
CDF
K
Motif Peak Velocity
20Hz
40Hz

**Figure S3. Rebound reaching following SNr inhibition exhibits enhanced kinematic signatures, related to Figure 2.**

**(A)** Schematic of variable-duration optogenetics inhibition. A 473 nm laser was delivered at water delivery onset for randomly interleaved durations (1 s, 2 s, 3 s, 4 s). The motif color map (inset) applies to all panels.
**(B)** Motif ethograms aligned to laser onset (left) and the laser offset (right), stratified by inhibition duration in descending order. Shaded regions indicate laser-on periods (n = 808 trials, 14 sessions,5 mice).
**(C)** Quantification of reach probability during (Within Laser Reach Probability) and immediately following (After Laser Reach Probability) inhibition (n = 14 sessions, 5 mice).
**(D)** Cumulative Distribution Function (CDF) plots of reach latency of all trials, stratified by inhibition duration (dark to light purple) compared to controls (orange) (n = 1,652 control and 808 stimulation trials; 14 sessions, 5 mice).
**(E)** Motif transition matrices. Left: reaching in control trials; right: rebound reaching in stimulation trials; middle: difference between rebound and control transition matrices. (n = 1,652 control trials and 808 stimulation trials from 14 sessions, 5 mice).
**(F and G)** Comparison of reach success probability **(F)** and latency to success **(G)** between control and rebound reaches (n = 14 sessions, 4 mice; Wilcoxon Signed-Rank Test).
**(H)** Superimposed motif ethograms aligned to the first Lift motif, comparing rebound (saturated colors) and control (lighter colors) reaches (n = 1,652 control and 808 stimulation trials; 14 sessions, 5 mice).
**(I)** Session average forelimb velocity profiles (mean ± SEM) aligned to the onset of Lift, Reach, Open, Grasp, and Retract motifs (left to right). Orange lines: control reaches; Purple lines: rebound reaches. (n = 14 sessions, 4 mice).
**(J and K)** Quantification of motif Mean Velocity **(J)** and Peak Velocity **(K)** for control vs rebound reaches. Top panels: Cumulative Distribution Function (CDF) (n = 1,652 control and 808 stimulation trials; 14 sessions, 5 mice); bottom panels: violin plots (n = 12 sessions, 4 mice; Wilcoxon Signed-Rank Test). Dots in the bar and violin plots represent individual session means.

Data in bar plot are shown as mean ± SEM. Dots in the bar and violin plots represent individual session means. Violin plots display the full data distribution density; internal box plots represent the median (center line) and the interquartile range. ns no significance, $*p < 0.05$, $**p < 0.01$, $***p < 0.001$, $****p < 0.0001$.

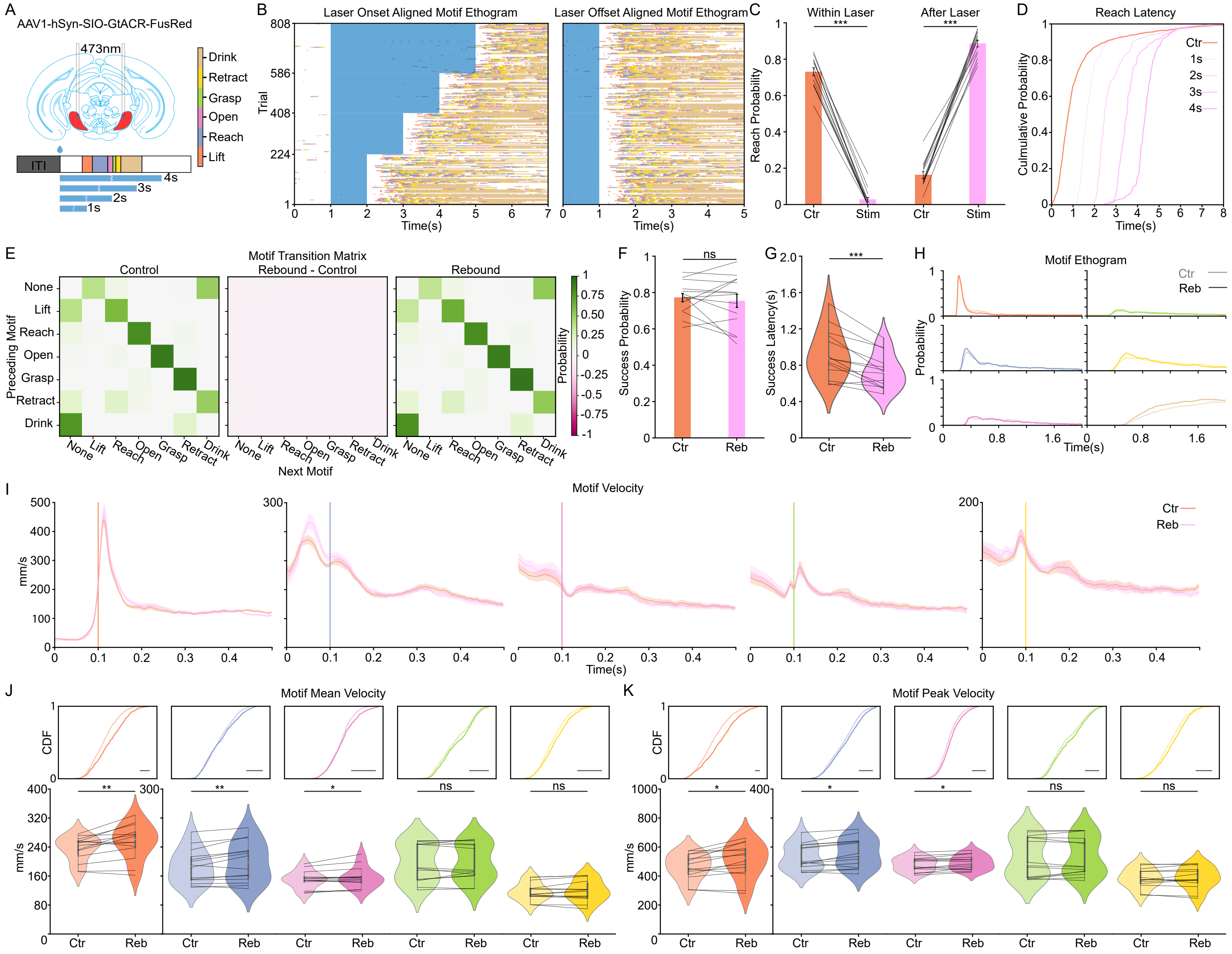

A
AAV1-hSyn-SIO-GtACR-FusRed
473nm
Drink
Retract
Grasp
Open
Reach
Lift
ITI
4s
3s
2s
1s
B
Laser Onset Aligned Motif Ethogram
Laser Offset Aligned Motif Ethogram
Trial
Time(s)
C
Within Laser
After Laser
Reach Probability
Ctr
Stim
D
Reach Latency
Culmulative Probability
Time(s)
E
Motif Transition Matrix
Control
Rebound - Control
Rebound
Preceding Motif
Next Motif
None
Lift
Reach
Open
Grasp
Retract
Drink
Probability
F
Success Probability
ns
G
Success Latency(s)
H
Motif Ethogram
Probability
Ctr
Reb
I
Motif Velocity
mm/s
Time(s)
J
Motif Mean Velocity
CDF
K
Motif Peak Velocity

**Figure S4. Frequency-dependent modulation of forelimb reaching behavior by SNr photoactivation, related to Figure 3.**

**(A)** Schematic of the optogenetic activation strategy. An excitatory opsin (AAV5-EF1a-DIO-ChR2-eYFP) was injected into the SNr and an optical fiber was implanted above the region. Photoactivation was delivered using a 473 nm blue laser at two different frequencies (20Hz, 40Hz), applied randomly across trials starting at water delivery and lasting for 4 seconds. The motif color map (inset) is used throughout the figure.

**(B–D)** Quantification of reach initiation dynamics: **(B)** Probability of reaching during laser delivery (Within Laser Reach Probability); **(C)** Probability of reaching following laser cessation (After Laser Reach Probability); and **(D)** Latency to Reach. (n = 12 sessions, 4 mice; Wilcoxon Signed-Rank Test).

**(E and F)** Quantification of reach success parameters: **(E)** Probability of success during laser delivery (Within Laser Success Probability); **(F)** Latency to success from reaching during laser delivery (Latency to Success). (n = 12 sessions, 4 mice; Wilcoxon Signed-Rank Test).

**(G)** Motif transition matrix difference maps, stratified by laser frequency. Each matrix displays the difference in transition probability (Activation - Control). n = 12 sessions, 4 mice.

**(H)** Motif ethogram aligned to the first Lift motif, stratified by inhibition intensity (n = 12 sessions, 4 mice).

**(I)** Session average forelimb velocity profiles (mean ± SEM) aligned to the onset of Lift, Reach, Open, Grasp, and Retract motifs (left to right). Orange lines represent control trials, and purple lines represent activation trials.

**(J and K)** Quantification of motif Mean Velocity and Peak Velocity across different laser frequencies. Top panels show the Cumulative Distribution Function (CDF) plots; bottom panels show the violin plots. (n = 12 sessions, 4 mice; Wilcoxon Signed-Rank Test).

Data in bar plot are shown as mean ± SEM. Dots in the bar and violin plots represent individual session means. Violin plots display the full data distribution density; internal box plots represent the median (center line) and the interquartile range. ns no significance, *p < 0.05, **p < 0.01, ***p < 0.001, ****p < 0.0001.

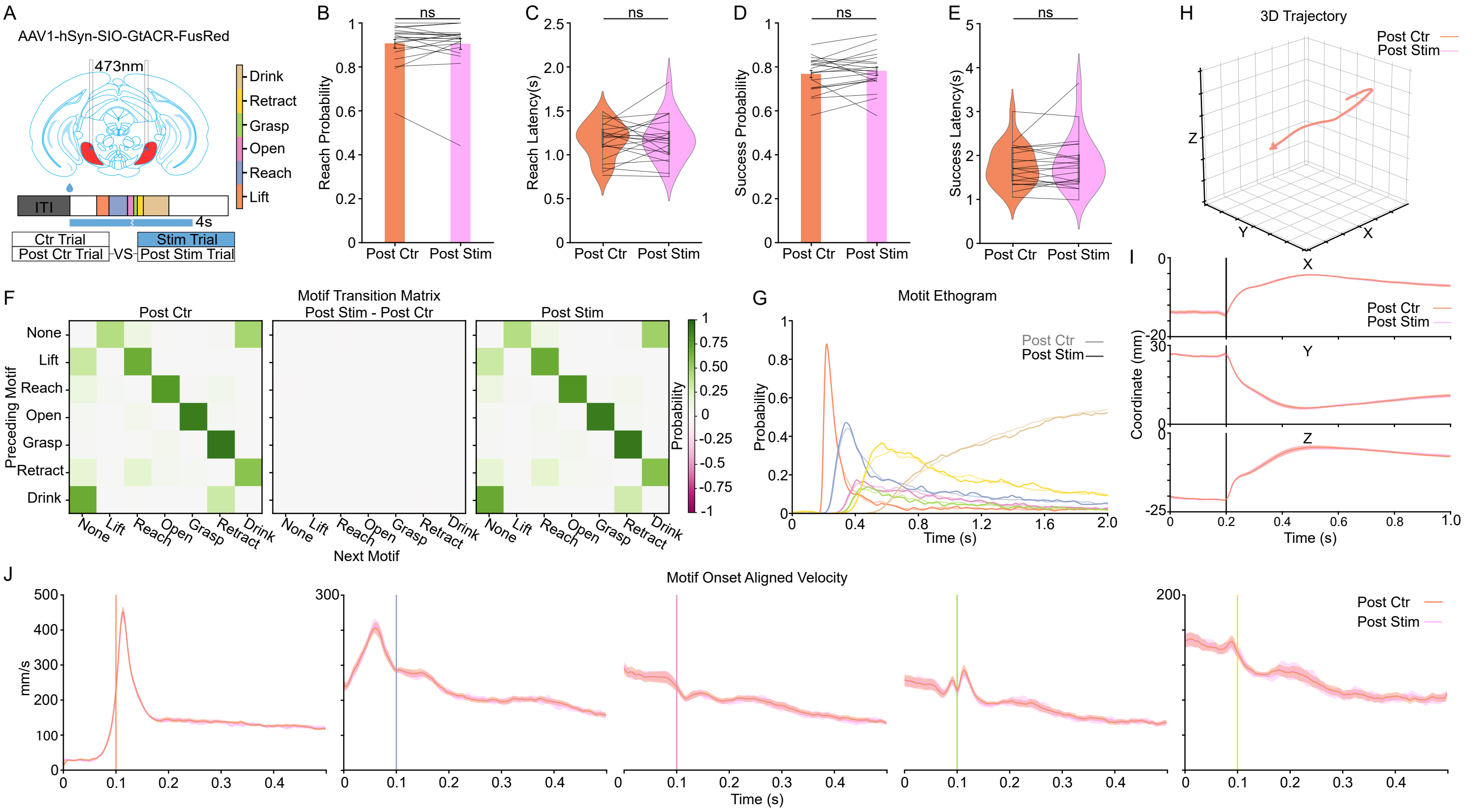

A
AAV1-hSyn-SIO-GtACR-FusRed
473nm
Drink
Retract
Grasp
Open
Reach
Lift
ITI
4s
Ctr Trial
Stim Trial
Post Ctr Trial
VS
Post Stim Trial
B
ns
Reach Probability
Post Ctr
Post Stim
C
ns
Reach Latency(s)
D
ns
Success Probability
E
ns
Success Latency(s)
H
3D Trajectory
Post Ctr
Post Stim
F
Motif Transition Matrix
Post Ctr
Post Stim - Post Ctr
Post Stim
Preceding Motif
None
Lift
Reach
Open
Grasp
Retract
Drink
Next Motif
Probability
G
Motit Ethogram
Post Ctr
Post Stim
Probability
Time (s)
I
Coordinate (mm)
X
Y
Z
Time (s)
J
Motif Onset Aligned Velocity
mm/s
Time (s)
Post Ctr
Post Stim

**Figure S5**. **SNr photoinhibition does not produce carryover effects on subsequent control trials, related to Figure 2.**

**(A)** Schematic of the experimental strategy to assess carryover effects. Top: Viral expression and fiber implantation in the SNr. Middle: Diagram of the trial-based reaching task and optogenetics protocol. Bottom: Definition of trial categories; Post Ctr denotes control trials immediately following a control trial, and Post Stim denotes control trials immediately following a stimulation trial. The motif color map (inset) applies to all panels.

**(B–E)** Quantification of reach dynamics comparing Post Ctr and Post Stim conditions: **(B)** Probability of reaching; **(C)** Latency to Reach; **(D)** Probability of success; and **(E)** Latency to Success. Data in bar plot are shown as mean ± SEM. Dots in the bar and violin plots represent individual session means. Violin plots display the full data distribution density; internal box plots represent the median (center line) and the interquartile range. No significant differences were observed (n = 21 sessions, 7 mice; Wilcoxon Signed-Rank Test).

**(F)** Motif transition matrices for Post Ctr Trials (left) and Post Stim Trials (right). The difference matrix (middle) indicates no substantial deviation in motif sequence progression (n = 1,917 Post Ctr and 770 Post Stim Trials; 21 sessions, 7 mice).

**(G)** Superimposed motif ethograms aligned to the first Lift motif, comparing Post Ctr (lighter colors) and Post Stim (saturated colors) Trials (n = 1,917 Post Ctr Trials and 770 Post Stim Trials from 21 sessions, 7 mice.

**(H and I)** Forelimb reaching kinematics aligned to the first Lift motif: **(H)** Average 3D forelimb trajectories and **(I)** corresponding X, Y, Z coordinate traces. Orange lines: Post Ctr; Pink lines: Post Stim (n = 21 sessions, 7 mice).

**(J)** Session average forelimb velocity profiles (mean ± SEM) aligned to the onset of Lift, Reach, Open, Grasp, and Retract motifs (left to right). Orange lines represent control trials, and purple lines represent activation trials. (n = 21 sessions, 7 mice).

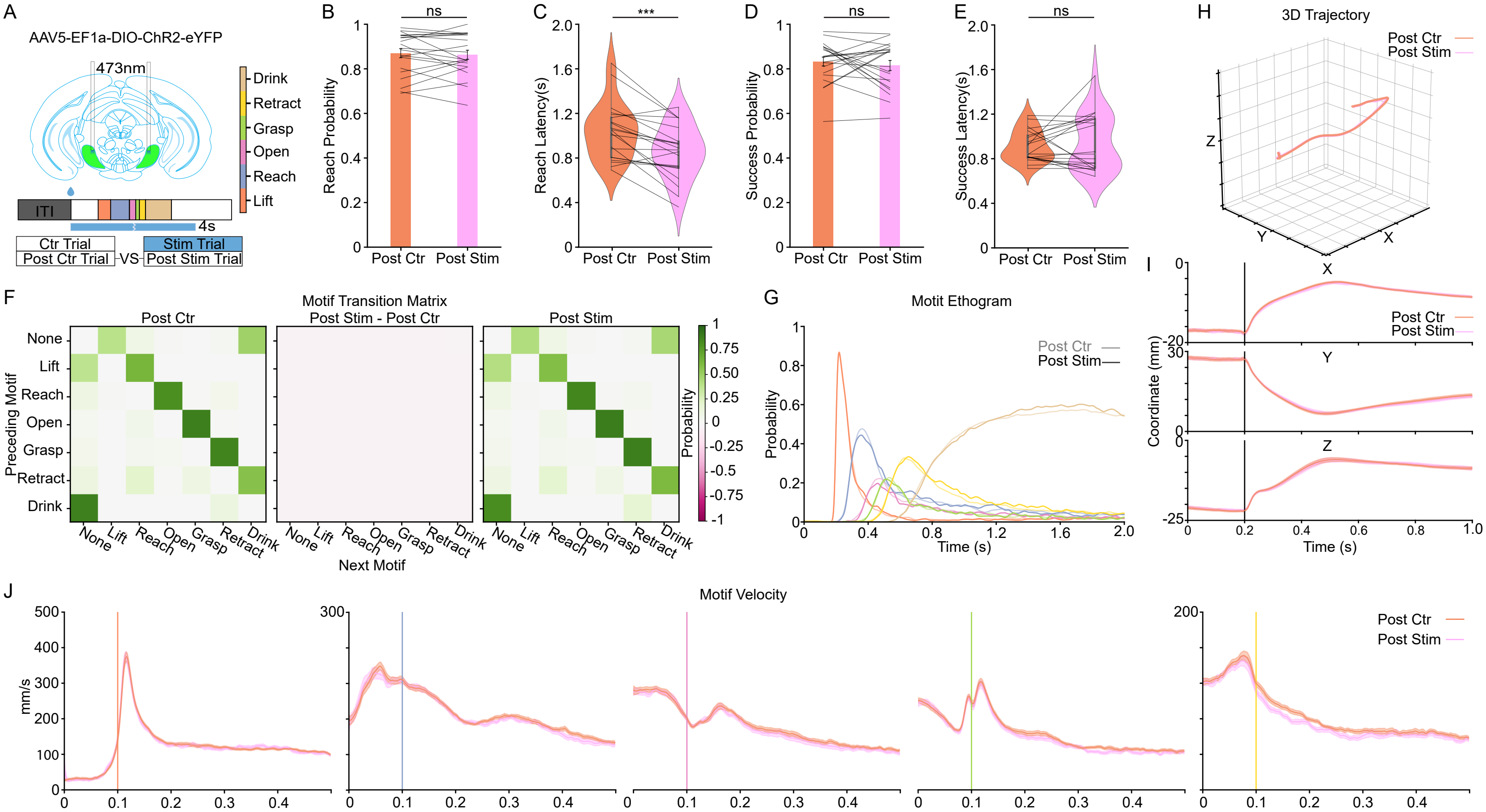
A
AAV5-EF1a-DIO-ChR2-eYFP
473nm
ITI
4s
Ctr Trial
Stim Trial
Post Ctr Trial
VS
Post Stim Trial
Drink
Retract
Grasp
Open
Reach
Lift
B
Reach Probability
ns
Post Ctr
Post Stim
C
Reach Latency(s)
***
D
Success Probability
ns
E
Success Latency(s)
ns
H
3D Trajectory
Post Ctr
Post Stim
F
Motif Transition Matrix
Post Ctr
Post Stim - Post Ctr
Post Stim
Preceding Motif
Next Motif
None
Lift
Reach
Open
Grasp
Retract
Drink
Probability
G
Motit Ethogram
Probability
Time (s)
I
Coordinate (mm)
Time (s)
J
Motif Velocity
mm/s

**Figure S6. SNr photoactivation does not produce carryover effects on subsequent control trials, related to Figure 3.**

**(A)** Schematic of the experimental strategy to assess carryover effects. Top: Viral expression and fiber implantation in the SNr. Middle: Diagram of the trial-based reaching task and optogenetics protocol. Bottom: Definition of trial categories; Post Ctr denotes control trials immediately following a control trial, and Post Stim denotes control trials immediately following a stimulation trial. The motif color map (inset) applies to all panels.

**(B–E)** Quantification of reach dynamics comparing Post Ctr and Post Stim conditions: **(B)** Probability of reaching; **(C)** Latency to Reach; **(D)** Probability of success; and **(E)** Latency to Success. Data in bar plot are shown as mean ± SEM. Dots in the bar and violin plots represent individual session means. Violin plots display the full data distribution density; internal box plots represent the median (center line) and the interquartile range. (n = 12 sessions, 4 mice; Wilcoxon Signed-Rank Test). ns no significance, $^{*}p < 0.05$, $^{**}p < 0.01$, $^{***}p < 0.001$, $^{****}p < 0.0001$.

**(F)** Motif transition matrices for Post Ctr Trials (left) and Post Stim Trials (right). The difference matrix (middle) indicates no substantial deviation in motif sequence progression (n = 1,320 Post Ctr and 504 Post Stim Trials; 14 sessions, 4 mice).

**(G)** Superimposed motif ethograms aligned to the first Lift motif, comparing Post Ctr (lighter colors) and Post Stim (saturated colors) Trials (n = 1, 320 Post Ctr Trials and 504 Post Stim Trials from 14 sessions, 4 mice.

**(H and I)** Forelimb reaching kinematics aligned to the first Lift motif: **(H)** Average 3D forelimb trajectories and **(I)** corresponding X, Y, Z coordinate traces. Orange: Post Ctr; Purple: Post Stim (n = 14 sessions, 4 mice).

**(J)** Session average forelimb velocity profiles (mean ± SEM) aligned to the onset of Lift, Reach, Open, Grasp, and Retract motifs (left to right). Orange lines represent control trials, and purple lines represent activation trials. (n = 14 sessions, 4 mice).

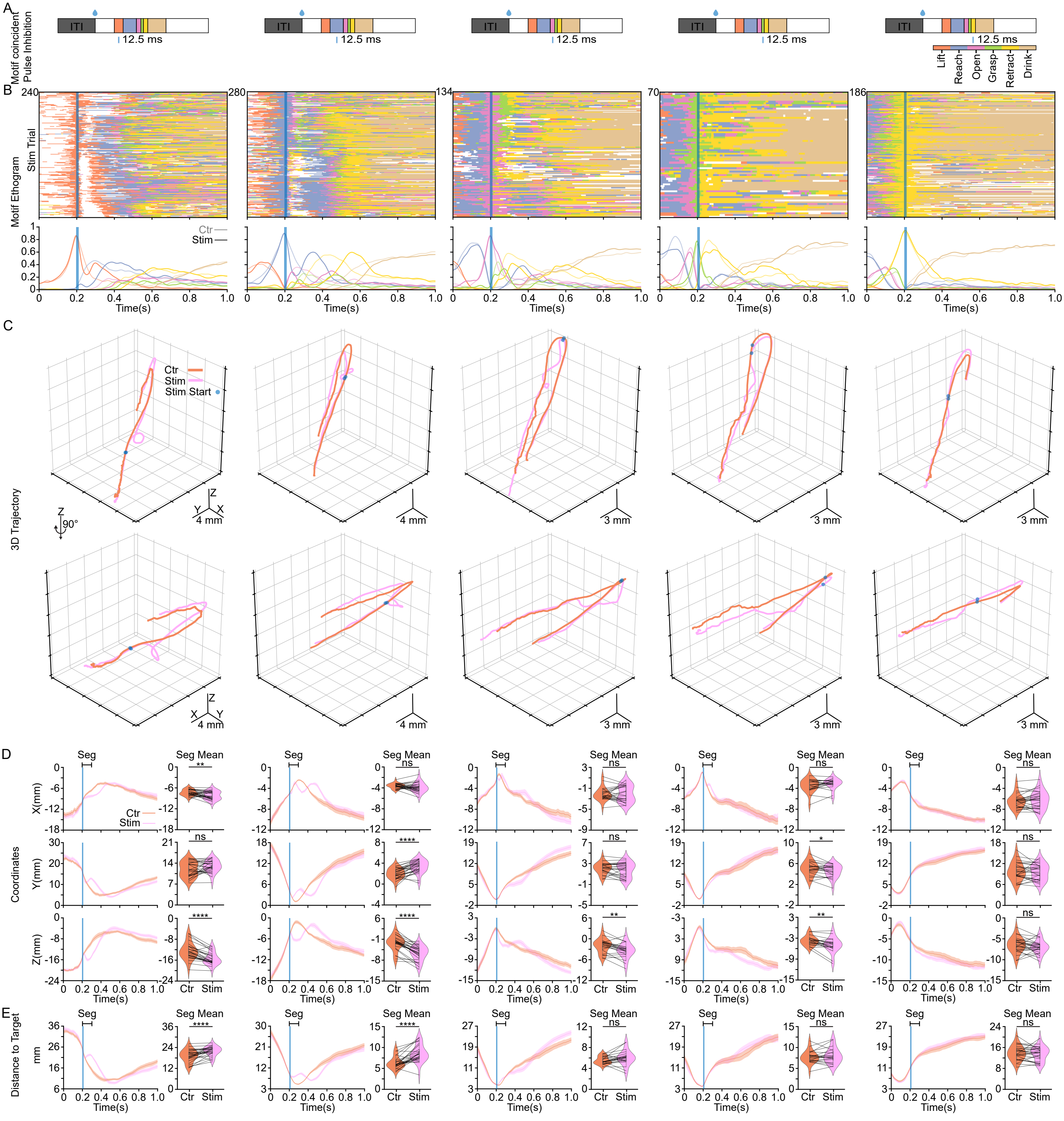

A
Motif coincident Pulse Inhibition
ITI
12.5 ms
Lift
Reach
Open
Grasp
Retract
Drink
B
Motif Ethogram
Stim Trial
Ctr
Stim
Time(s)
C
3D Trajectory
Ctr
Stim
Stim Start
Z
Y
X
4 mm
3 mm
90°
D
Coordinates
X(mm)
Y(mm)
Z(mm)
Seg
Seg Mean
ns
*
**
****
E
Distance to Target
mm

**Figure S7. Brief SNr pulse photoinhibition produces real-time modulation of reaching kinematics, related to Figure 4.**

**(A)** Schematic of the experimental strategy. A single 12.5 ms photoinhibition pulse was delivered at a randomized time after water delivery onset. Trials were grouped and analyzed post-hoc based on the action motif coincident with pulse onset (Lift, Reach, Open, Grasp, or Retract). The motif color map (inset) applies to all panels.

**(B)** Motif ethograms (top) and corresponding probability (bottom) aligned to laser pulse onset, stratified by the coincident motif (left to right: Lift, Reach, Open, Grasp, or Retract). The blue-shaded region indicates the laser duration. (Total n = 898 trials from 26 sessions, 7 mice; Lift: 240, Reach: 280, Open: 134, Grasp: 70, Retract: 186 trials).

**(C)** Average 3D forelimb trajectories aligned to laser pulse onset (blue dots), stratified by the coincident motif. Orange lines: control trials; Purple lines: stimulation trials. (n = 26 sessions, 7 mice).

**(D)** Forelimb coordinates (X, Y, Z) aligned to laser pulse onset. Left: temporal traces (mean ± SEM). Right: paired comparison of mean coordinates averaged over a 100 ms window starting at laser onset (Seg). Violin plots display the full data distribution density; internal box plots represent the median (center line) and the interquartile range. Orange: control trials; Purple: stimulation trials. (n = 26 sessions, 7 mice; Wilcoxon Signed-Rank Test). ns no significance, *p < 0.05, **p < 0.01, ***p < 0.001, ****p < 0.0001.

**(E)** Forelimb distance to target aligned to laser pulse onset. Left: temporal traces (mean ± SEM). Right: paired comparison of mean distance averaged over a 100 ms window starting at laser onset (Seg). Violin plots display the full data distribution density; internal box plots represent the median (center line) and the interquartile range. Orange: control trials; Purple: stimulation trials. (n = 26 sessions, 7 mice; Wilcoxon Signed-Rank Test). ns no significance, *p < 0.05, **p < 0.01, ***p < 0.001, ****p < 0.0001.

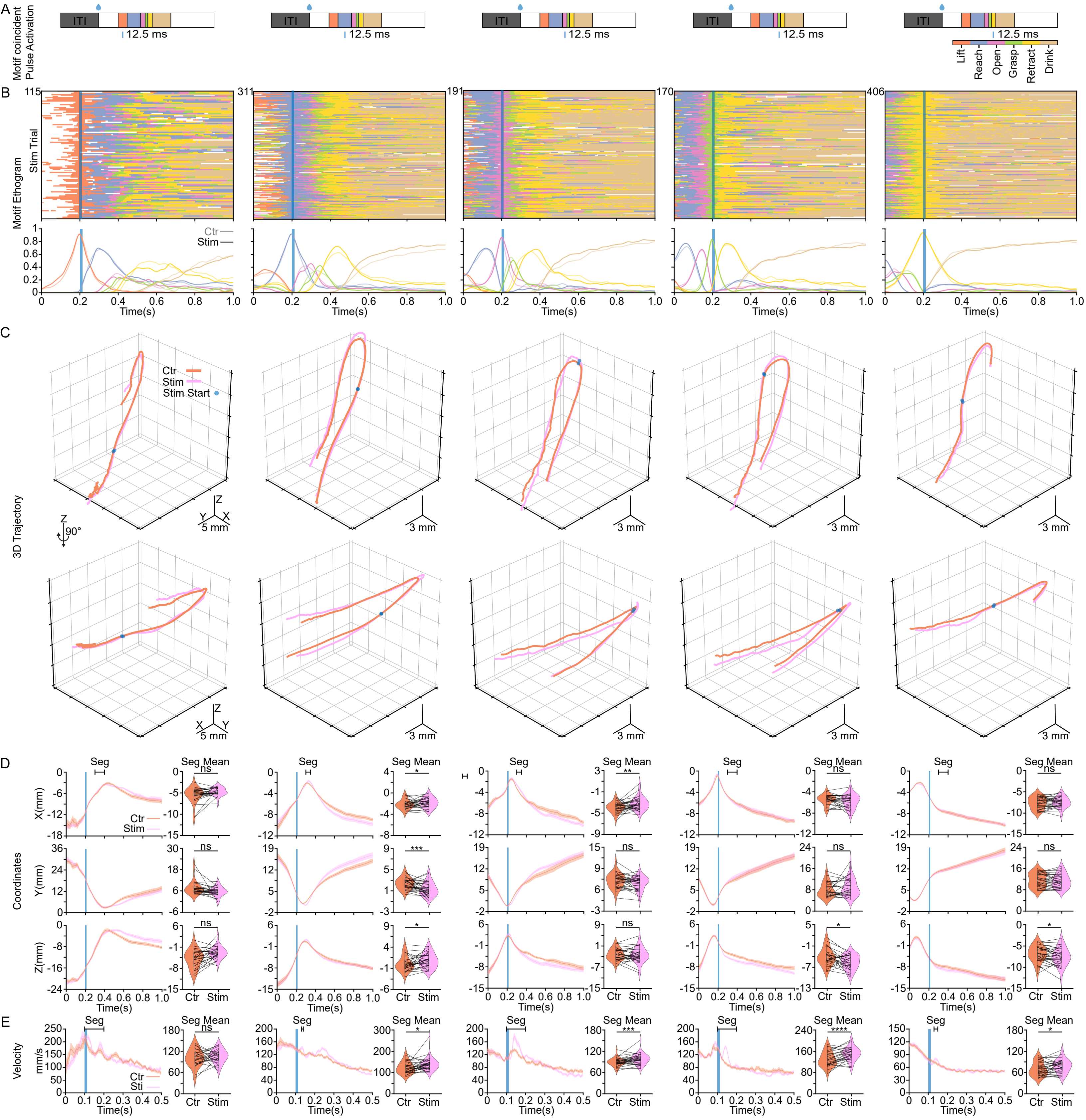
A
Motif coincident Pulse Activation
ITI
12.5 ms
Lift
Reach
Open
Grasp
Retract
Drink
B
Motif Ethogram
Stim Trial
115
311
191
170
406
Ctr
Stim
Time(s)
C
3D Trajectory
Ctr
Stim
Stim Start
Z
Y
X
5 mm
3 mm
90°
D
Coordinates
X(mm)
Y(mm)
Z(mm)
Seg
Seg Mean
ns
*
**
***
****
Ctr
Stim
Time(s)
E
Velocity
mm/s
Sti

**Figure S8. Brief SNr pulse photoactivation produces real-time modulation of reaching kinematics, related to Figure 5.**

**(A)** Schematic of the experimental strategy. A single 12.5 ms photoinhibition pulse was delivered at a randomized time after water delivery onset. Trials were grouped and analyzed post-hoc based on the action motif coincident with pulse onset (Lift, Reach, Open, Grasp, or Retract). The motif color map (inset) applies to all panels.

**(B)** Motif ethograms (top) and corresponding probability (bottom) aligned to laser pulse onset, stratified by the coincident motif (left to right: Lift, Reach, Open, Grasp, or Retract). The blue-shaded region indicates the laser duration. (Total n = 1193 trials from 30 sessions, 4 mice; Lift: 115, Reach: 311, Open: 191, Grasp: 170, Retract: 406 trials).

**(C)** Average 3D forelimb trajectories aligned to laser pulse onset (blue dots), stratified by the coincident motif. Orange lines: control trials; Purple lines: stimulation trials. (n = 30 sessions, 4 mice).

**(D)** Forelimb coordinates (X, Y, Z) aligned to laser pulse onset. Left: temporal traces (mean ± SEM). Right: paired comparison of mean coordinates averaged over a defined window (Seg). Violin plots display the full data distribution density; internal box plots represent the median (center line) and the interquartile range. Orange: control trials; Purple: stimulation trials. (n = 30 sessions, 4 mice; Wilcoxon Signed-Rank Test). ns no significance, *$p < 0.05$, **$p < 0.01$, ***$p < 0.001$, ****$p < 0.0001$.

**(E)** Forelimb velocity aligned to laser pulse onset. Left: temporal traces (mean ± SEM). Right: paired comparison of mean velocity averaged over a defined window (Seg). Violin plots display the full data distribution density; internal box plots represent the median (center line) and the interquartile range. Orange: control trials; Purple: stimulation trials. (n = 30 sessions, 4 mice; Wilcoxon Signed-Rank Test). ns no significance, *$p < 0.05$, **$p < 0.01$, ***$p < 0.001$, ****$p < 0.0001$.

**Supplementary Video 1. Reaching task structure and skilled forelimb motifs.** Example trial demonstrating the behavioral setup, task progression, and motor motifs in the skilled forelimb reaching task. Related to Figure 1.

**Supplementary Video 2. 3D trajectory reconstructions of forelimb reaching motifs.** Reconstructed 3D trajectories of all trials from an example session, color-coded by action motif. The video shows 500 total frames per trial, beginning 100 frames prior to the first Grasp motif following water delivery. Trajectory tails are set to a 50-frame fade. Related to Figure 1.

**Supplementary Video 3. Behavioral effects of SNr manipulation.** Representative trials showing the effects of 4-s optogenetic inhibition, chemoinhibition, 4-s optogenetic activation, and chemoactivation on forelimb skilled behavior. Related to Figures 2 and 3.

**Supplementary Video 4. Session-averaged 3D trajectories under SNr manipulation.** Reconstructed 3D trajectories (session averages) comparing experimental (purple) and control (orange) conditions. Data includes 4-s optogenetic inhibition, chemoinhibition, 4-s optogenetic activation, and chemoactivation. Each curve represents a single session. Video shows 250 frames per session, starting 50 frames prior to laser onset. Related to Figures 2 and 3.

**Supplementary Video 5. Behavioral effects of short optogenetic manipulation.** Example trials demonstrating the effects of short optogenetics manipulation coinciding with specific motor motifs: 100-ms and 12.5-ms inhibition coinciding with Lift; 100-ms and 12.5-ms inhibition coinciding with Reach; and 300-ms and 12.5-ms activation coinciding with Reach. Related to Figures 4 and 5.

**Supplementary Video 6. Reconstructed 3D trajectories under short optogenetic manipulation.** 3D trajectories of all trials from representative mice during short optogenetic manipulations. Includes 100-ms and 12.5-ms inhibition (Lift and Reach) and 300-ms and 12.5-ms activation (Lift-to-Retract). Purple: Inhibition; Orange: Control. Each curve represents one trial color-coded by action motif. Video shows 250 frames per trial, starting 50 frames prior to laser onset. Related to Figures 4 and 5.

**Supplementary Table 1. Spearman's rank correlation coefficient of Motif specific population calcium Zscore and forelimb movement kinematics, related to Figure 1.**

| | Ipsilateral | | Contralateral | | Ipsilateral | | | | Contralateral | | | |
|---|---|---|---|---|---|---|---|---|---|---|---|---|
| Zscore | Mean | Peak | Mean | Peak | Mean | Peak | Mean | Peak | Mean | Peak | Mean | Peak |
| | Motif Trajectory Length | | | | Vm | Vp | Vm | Vp | Vm | Vp | Vm | Vp |
| Lift | R: 0.140 p: 0.544 | R: 0.110 p: 0.634 | R: 0.061 p: 0.793 | R: 0.090 p: 0.695 | R: -0.349 p: 0.121 | R: -0.238 p: 0.300 | R: -0.347 p: 0.124 | R: -0.258 p: 0.258 | R: -0.266 p: 0.243 | R: -0.230 p: 0.316 | R: -0.260 p: 0.256 | R: -0.206 p: 0.369 |
| Reach | R: 0.105 p: 0.650 | R: 0.049 p: 0.832 | R: 0.404 p: 0.069 | R: 0.518 p: 0.016 | R: 0.208 p: 0.366 | R: 0.119 p: 0.606 | R: 0.114 p: 0.622 | R: 0.057 p: 0.806 | R: 0.300 p: 0.186 | R: 0.401 p: 0.071 | R: 0.390 p: 0.081 | R: 0.505 p: 0.019 |
| Open | R: 0.526 p: 0.014 | R: 0.526 p: 0.014 | R: 0.560 p: 0.008 | R: 0.588 p: 0.005 | R: 0.496 p: 0.022 | R: 0.553 p: 0.009 | R: 0.479 p: 0.028 | R: 0.547 p: 0.010 | R: 0.409 p: 0.066 | R: 0.617 p: 0.003 | R: 0.436 p: 0.048 | R: 0.638 p: 0.002 |
| Grasp | R: 0 .009 p: 0.969 | R: -0.032 p: 0.889 | R: 0.591 p: 0.005 | R: 0.604 p: 0.004 | R: 0.406 p: 0.067 | R: 0.390 p: 0.081 | R: 0.365 p: 0.104 | R: 0.348 p: 0.122 | R: 0.573 p: 0.007 | R: 0.562 p: 0.008 | R: 0.577 p: 0.006 | R: 0.562 p: 0.008 |
| Retract | R: 0.094 p: 0.687 | R: 0.103 p: 0.658 | R: 0.625 p: 0.002 | R: 0.630 p: 0.002 | R: 0.503 p: 0.020 | R: 0.291 p: 0.201 | R: 0.509 p: 0.018 | R: 0.294 p: 0.197 | R: 0.622 p: 0.003 | R: 0.504 p: 0.020 | R: 0.622 p: 0.003 | R: 0.512 p: 0.018 |

**Supplementary Table 2. Statistics summary table.**

| Fig | Statistic Method | Sample Size | P value |
|---|---|---|---|
| 1I | Spearman's rank correlation coefficient | 21 sessions from 7 mice | **See Table 1** |
| 1J | Pearson correlation coefficient | 21 sessions from 7 mice | **Top:** R = 0.571, p = 0.007<br>**Bottom:** R = 0.658, p = 0.001 |
| 2B | Wilcoxon Signed-Rank Test | 21 sessions from 7 mice | < 0.0001 |
| 2C | Wilcoxon Signed-Rank Test | 21 sessions from 7 mice | 0.272 |
| 2E | Wilcoxon Signed-Rank Test | 21 sessions from 7 mice | **From left to right:**<br>< 0.0001 \| < 0.0001 \| < 0.0001<br>< 0.0001 \| < 0.0001 \| < 0.0001 |
| 2F | Wilcoxon Signed-Rank Test | 21 sessions from 7 mice | **From left to right:**<br>< 0.0001 \| < 0.0001 \| < 0.0001<br>< 0.0001 \| < 0.0001 \| 0.0002 |
| 2G | Wilcoxon Signed-Rank Test | 21 sessions from 7 mice | **From left to right:**<br>0.014 \| < 0.0001 \| < 0.0001<br>< 0.0001 \| < 0.0001 \| < 0.0001 |
| 2H | Wilcoxon Signed-Rank Test | 21 sessions from 7 mice | **From left to right:**<br>0.046 \| < 0.0001 \| 0.0002<br>< 0.0001 \| < 0.0001 \| < 0.0001 |
| 2J | Wilcoxon Signed-Rank Test | 21 sessions from 7 mice | **From left to right:**<br>0.0002 \| 0.0003 \| < 0.0001 \| < 0.0001 \| 0.070 |
| 2K | Wilcoxon Signed-Rank Test | 21 sessions from 7 mice | **From left to right:**<br>0.002 \| 0.0002 \| < 0.0001 \| < 0.0001 \| 0.035 |

| | | | |
|---|---|---|---|
| 2M | Wilcoxon Signed-Rank Test | 15 sessions from 5 mice | 0.389 |
| 2N | Wilcoxon Signed-Rank Test | 15 sessions from 5 mice | 0.0001 |
| 2P | Wilcoxon Signed-Rank Test | 15 sessions from 5 mice | **From left to right:**<br>0.598 \| 0.086 \| 0.124 \| 0.125 \| 0.098 \| 0.035 |
| 2Q | Wilcoxon Signed-Rank Test | 15 sessions from 5 mice | **From left to right:**<br>0.720 \| 0.359 \| 0.229 \| 0.303 \| 0.252 \| 0.022 |
| 2R | Wilcoxon Signed-Rank Test | 15 sessions from 5 mice | **From left to right:**<br>0.0004 \| 0.002 \| 0.004 \| 0.005 \| 0.002 \| 0.0001 |
| 2S | Wilcoxon Signed-Rank Test | 15 sessions from 5 mice | **From left to right:**<br>0.0001 \| 0.010 \| 0.048 \| 0.005 \| 0.012 \| 0.0004 |
| 2U | Wilcoxon Signed-Rank Test | 15 sessions from 5 mice | **From left to right:**<br>0.002 \| < 0.0001 \| 0.389 \| 0.083 \| 0.026 |
| 2V | Wilcoxon Signed-Rank Test | 15 sessions from 5 mice | **From left to right:**<br>< 0.0001 \| < 0.0001 \| 0.003 \| 0.018 \| 0.008 |
| 3B | Wilcoxon Signed-Rank Test | 21 sessions from 4 mice | 0.733 |
| 3C | Wilcoxon Signed-Rank Test | 21 sessions from 4 mice | < 0.0001 |
| 3E | Wilcoxon Signed-Rank Test | 21 sessions from 4 mice | **From left to right:**<br>0.683 \| 0.683 \| 0.683 \| 0.733 \| 0.865 \| 0.562 |
| 3F | Wilcoxon Signed-Rank Test | 21 sessions from 4 mice | **From left to right:**<br>0.393 \| 0.203 \| 0.035 \| 0.179 \| 0.320 \| 0.065 |
| 3G | Wilcoxon Signed-Rank Test | 21 sessions from 4 mice | **From left to right:**<br>< 0.0001 \| < 0.0001 \| 0.229<br>0.946 \| 0.060 \| 0.168 |
| 3H | Wilcoxon Signed-Rank Test | 21 sessions from 4 mice | **From left to right:**<br>0.006 \| 0.243 \| 0.002 \| 0.016 \| 0.355 \| 0.096 |
| 3J | Wilcoxon Signed-Rank Test | 21 sessions from 4 mice | **From left to right:**<br>0.018 \| 0.002 \| 0.119 \| 0.007 \| 0.004 |
| 3K | Wilcoxon Signed-Rank Test | 21 sessions from 4 mice | **From left to right:**<br>0.272 \| 0.0006 \| 0.006 \| 0.002 \| 0.0004 |
| 3M | Wilcoxon Signed-Rank Test | 12 sessions from 4 mice | 0.110 |
| 3N | Wilcoxon Signed-Rank Test | 12 sessions from 4 mice | 0.007 |
| 3P | Wilcoxon Signed-Rank Test | 12 sessions from 4 mice | **From left to right:**<br>0.212 \| 0.306 \| 0.350 \| 0.266 \| 0.306 \| 0.358 |
| 3Q | Wilcoxon Signed-Rank Test | 12 sessions from 4 mice | **From left to right:**<br>0.970 \| 0.622 \| 0.301 \| 0.266 \| 0.380 \| 0.092 |
| 3R | Wilcoxon Signed-Rank Test | 12 sessions from 4 mice | **From left to right:**<br>0.016 \| 0.007 \| 0.569 \| 0.622 \| 0.005 \| 0.970 |
| 3S | Wilcoxon Signed-Rank Test | 12 sessions from 4 mice | **From left to right:**<br>0.470 \| 0.850 \| 0.003 \| 0.424 \| 0.151 \| 0.470 |
| 3U | Wilcoxon Signed-Rank Test | 12 sessions from 4 mice | **From left to right:**<br>0.005 \| 0.001 \| 0.005 \| 0.042 \| 0.002 |
| 3V | Wilcoxon Signed-Rank Test | 12 sessions from 4 mice | **From left to right:**<br>0.380 \| 0.001 \| 0.0015 \| 0.027 \| 0.027 |
| 4C | Wilcoxon Signed-Rank Test | 33 sessions from 7 mice | **Left to Right (Lift to Retract):**<br>0.0002 \| 0.0001 \| 0.016 \| 0.012 \| 0.009 |
| 4D | Wilcoxon Signed-Rank Test | 33 sessions from 7 mice | **Left to Right (Lift to Retract):**<br>0.012 \| 0.0001 \| 0.005 \| < 0.0001 \| < 0.0001 |
| 4F | Wilcoxon Signed-Rank Test | 33 sessions from 7 mice | **X Seg Mean:** 0.0002<br>**X Seg \|Peak\|:** < 0.0001 |

| | | | |
|---|---|---|---|
| | | | **Y Seg Mean:** 0.0004<br>**Y Seg \|Peak\|:** < 0.0001<br>**Z Seg Mean:** < 0.0001<br>**Z Seg \|Peak\|:** < 0.0001 |
| 4G | Wilcoxon Signed-Rank Test | 33 sessions from 7 mice | **Mean:** < 0.0001<br>**Peak:** < 0.0001 |
| 4H | Wilcoxon Signed-Rank Test | 33 sessions from 7 mice | **Mean:** < 0.0001<br>**Min:** < 0.0001 |
| 4K | Wilcoxon Signed-Rank Test | 26 sessions from 7 mice | **Left to Right (Lift to Retract):**<br>< 0.0001 \| < 0.0001 \| 0.002 \| 0.007 \| 0.012 |
| 4L | Wilcoxon Signed-Rank Test | 26 sessions from 7 mice | **Left to Right (Lift to Retract):**<br>0.005 \| < 0.0001 \| 0.277 \| 0.0001 \| 0.006 |
| 4N | Wilcoxon Signed-Rank Test | 26 sessions from 7 mice | **X Seg Mean:** 0.006<br>**X Seg \|Peak\|:** 0.006<br>**Y Seg Mean:** 0.025<br>**Y Seg \|Peak\|:** 0.089<br>**Z Seg Mean:** < 0.0001<br>**Z Seg \|Peak\|:** < 0.0001 |
| 4O | Wilcoxon Signed-Rank Test | 26 sessions from 7 mice | **Mean:** 0.0005<br>**Peak:** 0.012 |
| 4P | Wilcoxon Signed-Rank Test | 26 sessions from 7 mice | **Mean:** 0.014<br>**Min:** 0.0001 |
| 5D | Wilcoxon Signed-Rank Test | 13 sessions from 4 mice | **X Seg 1:** 0.048<br>**X Seg 2:** 0.017<br>**Y Seg 1:** 0.017<br>**Y Seg 2:** 0.027<br>**Z Seg 1:** 0.376<br>**Z Seg 2:** 0.001 |
| 5E | Wilcoxon Signed-Rank Test | 13 sessions from 4 mice | **Seg 1:** 0.027<br>**Seg 2:** 0.0007 |
| 5F | Wilcoxon Signed-Rank Test | 13 sessions from 4 mice | **Seg 1:** 0.0005<br>**Seg 2:** 0.001 |
| 5G | Wilcoxon Signed-Rank Test | 13 sessions from 4 mice | **Left to Right (Lift to Retract):**<br>0.970 \| 0.0002 \| 0.305 \| 0.002 \| 0.001 |
| 5H | Wilcoxon Signed-Rank Test | 13 sessions from 4 mice | **Left to Right (Lift to Retract):**<br>0.380 \| 0.0002 \| 0.216 \| 0.005 \| 0.001 |
| 5L | Wilcoxon Signed-Rank Test | 30 sessions from 4 mice | **X Seg 1:** 0.019<br>**X Seg 2:** 0.503<br>**Y Seg 1:** 0.015<br>**Y Seg 2:** 0.360<br>**Z Seg 1:** 0.903<br>**Z Seg 2:** 0.198 |
| 5M | Wilcoxon Signed-Rank Test | 30 sessions from 4 mice | **Seg 1:** 0.040<br>**Seg 2:** 0.221 |
| 5N | Wilcoxon Signed-Rank Test | 30 sessions from 4 mice | **Seg 1:** 0.004<br>**Seg 2:** 0.919 |
| 5O | Wilcoxon Signed-Rank Test | 30 sessions from 4 mice | **Left to Right (Lift to Retract):**<br>0.784 \| 0.382 \| 0.077 \| 0.005 \| 0.114 |
| 5P | Wilcoxon Signed-Rank Test | 30 sessions from 4 mice | **Left to Right (Lift to Retract):**<br>0.571 \| 0.584 \| 0.003 \| 0.008 \| 0.008 |
| S1B | Friedman test | 251 sessions from 28 mice | < 0.0001 |
| S1C | Friedman test | 251 sessions from 28 mice | < 0.0001 |

| | | | |
|---|---|---|---|
| S1D | Friedman test | 251 sessions from 28 mice | < 0.0001 |
| S1E | Friedman test | 251 sessions from 28 mice | < 0.0001 |
| S1F | Friedman test | 251 sessions from 28 mice | < 0.0001 |
| S1G | Friedman test | 251 sessions from 28 mice | < 0.0001 |
| S1H | Friedman test | 251 sessions from 28 mice | < 0.0001 |
| S1I | Friedman test | 251 sessions from 28 mice | < 0.0001 |
| S1J | Friedman test | 251 sessions from 28 mice | < 0.0001 |
| S1K | Friedman test | 251 sessions from 28 mice | < 0.0001 |
| S1L | Friedman test | 251 sessions from 28 mice | < 0.0001 |
| S1M | Friedman test | 251 sessions from 28 mice | < 0.0001 |
| S2B | Friedman test | 21 sessions from 7 mice | < 0.0001 |
| S2C | Friedman test | 21 sessions from 7 mice | < 0.0001 |
| S2D | Friedman test | 21 sessions from 7 mice | < 0.0001 |
| S2H | Friedman test | 19 sessions from 7 mice | < 0.0001 \| < 0.0001 \| < 0.0001 \| 0.0005 \| 0.065 |
| S2I | Friedman test | 19 sessions from 7 mice | 0.018 \| 0.0003 \| 0.0004 \| < 0.0001 \| 0.029 |
| S3C | Wilcoxon Signed-Rank Test | 14 sessions from 5 mice | **Within Laser:**<br>0.0001<br>**After Laser:**<br>0.0001 |
| S3F | Wilcoxon Signed-Rank Test | 14 sessions from 5 mice | 0.583 |
| S3G | Wilcoxon Signed-Rank Test | 14 sessions from 5 mice | 0.0004 |
| S3J | Wilcoxon Signed-Rank Test | 14 sessions from 5 mice | **Left to right:**<br>0.009 \| 0.007 \| 0.042 \| 1.000 \| 0.626 |
| S3K | Wilcoxon Signed-Rank Test | 14 sessions from 5 mice | **Left to right:**<br>0.013 \| 0.011 \| 0.013 \| 0.855 \| 0.626 |
| S4B | Wilcoxon Signed-Rank Test | 12 sessions from 4 mice | **20Hz - Control vs 40Hz - Control:** 0.301<br>**20 Hz vs Control:** 0.424<br>**40Hz vs Control:** 0.339 |
| S4C | Wilcoxon Signed-Rank Test | 12 sessions from 4 mice | **20Hz- Control vs 40Hz - Control:**<br>0.722<br>**20 Hz vs Control:**0.970<br>**40Hz vs Control:** 0.569 |
| S4D | Wilcoxon Signed-Rank Test | 12 sessions from 4 mice | **20Hz - Control vs 40Hz - Control** 0.233<br>**20 Hz vs Control:**0.910<br>**40Hz vs Control:** 0.110 |
| S4E | Wilcoxon Signed-Rank Test | 12 sessions from 4 mice | **20Hz - Control vs 40Hz - Control:** 0.077<br>**20 Hz vs Control:** 0.027<br>**40Hz vs Control:** 0.151 |

| | | | |
|---|---|---|---|
| S4F | Wilcoxon Signed-Rank Test | 12 sessions from 4 mice | **20Hz - Control vs 40Hz - Control:** 0.970<br>**20 Hz vs Control:** 0.001<br>**40Hz vs Control:** 0.007 |
| S4J | Wilcoxon Signed-Rank Test | 12 sessions from 4 mice | **20Hz- Control vs 40Hz-Control**:<br>0.233 \| 0.002 \| 0.677 \| 0.077 \| 0.204<br>**20 Hz vs Control:**<br>0.129 \| 0.034 \| 0.176 \| 0.077 \| 0.034<br>**40 Hz vs Control:**<br>0.064 \| 0.002 \| 0.110 \| 0.009 \| 0.009 |
| S4K | Wilcoxon Signed-Rank Test | 12 sessions from 4 mice | **20Hz- Control vs 40Hz-Control**:<br>1.000 \| 0.012 \| 0.110 \| 0.034 \| 0.034<br>**20 Hz vs Control:**<br>0.129 \| 0.012 \| 0.266 \| 0.064 \| 0.027<br>**40 Hz vs Control:**<br>0.424 \| 0.007 \| 0.002 \| 0.005 \| 0.002 |
| S5B | Wilcoxon Signed-Rank Test | 21 sessions from 7 mice | 0.881 |
| S5C | Wilcoxon Signed-Rank Test | 21 sessions from 7 mice | 0.838 |
| S5D | Wilcoxon Signed-Rank Test | 21 sessions from 7 mice | 0.683 |
| S5E | Wilcoxon Signed-Rank Test | 21 sessions from 7 mice | 0.157 |
| S6B | Wilcoxon Signed-Rank Test | 21 sessions from 7 mice | 0.708 |
| S6C | Wilcoxon Signed-Rank Test | 21 sessions from 7 mice | 0.0002 |
| S6D | Wilcoxon Signed-Rank Test | 21 sessions from 7 mice | 0.609 |
| S6E | Wilcoxon Signed-Rank Test | 21 sessions from 7 mice | 0.838 |
| S7D | Wilcoxon Signed-Rank Test | 26 sessions from 7 mice | **X Seg Mean:**<br>0.007 \| 0.143 \| 0.899 \| 0.216 \| 0.191<br>**Y Seg Mean:**<br>0.080 \| < 0.0001 \| 0.248 \| 0.027 \| 0.312<br>**Z Seg Mean:**<br>< 0.0001 \| < 0.0001 \| 0.002 \| 0.002 \| 0.120 |
| S7E | Wilcoxon Signed-Rank Test | 26 sessions from 7 mice | **Left to right:**<br>< 0.0001 \| < 0.0001 \| 0.085 \| 0.546 \| 0.634 |
| S8D | Wilcoxon Signed-Rank Test | 30 sessions from 4 mice | **X Seg Mean**<br>0.635 \| 0.038 \| 0.003 \| 0.324 \| 0.730<br>**Y Seg Mean**<br>0.898 \| 0.0005 \| 0.051 \| 0.324 \| 0.700<br>**Z Seg Mean**<br>0.689 \| 0.029 \| 0.701 \| 0.013 \| 0.038 |
| S8E | Wilcoxon Signed-Rank Test | 30 sessions from 4 mice | **Left to right:**<br>0.303 \| 0.019 \| 0.001 \| < 0.0001 \| 0.016 |
